# Association of *APOE* Genotypes and Chronic Traumatic Encephalopathy

**DOI:** 10.1001/jamaneurol.2022.1634

**Published:** 2022-06-27

**Authors:** Kathryn Atherton, Xudong Han, Jaeyoon Chung, Jonathan D. Cherry, Zachary Baucom, Nicole Saltiel, Evan Nair, Bobak Abdolmohammadi, Madeline Uretsky, Mohammed Muzamil Khan, Conor Shea, Shruti Durape, Brett M. Martin, Joseph N. Palmisano, Kurt Farrell, Christopher J. Nowinski, Victor E. Alvarez, Brigid Dwyer, Daniel H. Daneshvar, Douglas I. Katz, Lee E. Goldstein, Robert C. Cantu, Neil W. Kowall, Michael L. Alosco, Bertrand R. Huber, Yorghos Tripodis, John F. Crary, Lindsay Farrer, Robert A. Stern, Thor D. Stein, Ann C. McKee, Jesse Mez

**Affiliations:** 1 Boston University Bioinformatics Graduate Program, Boston, Massachusetts; 2 Boston University Alzheimer’s Disease and CTE Centers, Boston University School of Medicine, Boston, Massachusetts; 3 Department of Medicine (Biomedical Genetics), Boston University School of Medicine, Boston, Massachusetts; 4 VA Boston Healthcare System, Boston, Massachusetts; 5 Department of Pathology and Laboratory Medicine, Boston University School of Medicine, Boston, Massachusetts; 6 Department of Veterans Affairs Medical Center, Bedford, Massachusetts; 7 Boston University Department of Biostatistics, Boston University School of Public Health, Boston, Massachusetts; 8 Biostatistics & Epidemiology Data Analytics Center, Boston University School of Public Health, Boston, Massachusetts; 9 Department of Pathology, Fishberg Department of Neuroscience, Friedman Brain Institute, Ronald M. Loeb Center for Alzheimer’s Disease, Icahn School of Medicine at Mount Sinai, New York, New York; 10 Concussion Legacy Foundation, Boston, Massachusetts; 11 Braintree Rehabilitation Hospital, Braintree, Massachusetts; 12 Department of Neurology, Boston University School of Medicine, Boston, Massachusetts; 13 Department of Rehabilitation Medicine, Harvard Medical School, Boston, Massachusetts; 14 Department of Psychiatry, Boston University School of Medicine, Boston, Massachusetts; 15 Department of Neurosurgery, Emerson Hospital, Concord, Massachusetts

## Abstract

**Importance:**

Repetitive head impact (RHI) exposure is the chief risk factor for chronic traumatic encephalopathy (CTE). However, the occurrence and severity of CTE varies widely among those with similar RHI exposure. Limited evidence suggests that the *APOE*ε4 allele may confer risk for CTE, but previous studies were small with limited scope.

**Objective:**

To test the association between *APOE* genotype and CTE neuropathology and related endophenotypes.

**Design, Setting, and Participants:**

This cross-sectional genetic association study analyzed brain donors from February 2008 to August 2019 from the Veterans Affairs–Boston University–Concussion Legacy Foundation Brain Bank. All donors had exposure to RHI from contact sports or military service. All eligible donors were included. Analysis took place between June 2020 and April 2022.

**Exposures:**

One or more *APOE*ε4 or *APOE*ε2 alleles.

**Main Outcomes and Measures:**

CTE neuropathological status, CTE stage (0-IV), semiquantitative phosphorylated tau (p-tau) burden in 11 brain regions (0-3), quantitative p-tau burden in the dorsolateral frontal lobe (log-transformed AT8+ pixel count per mm^2^), and dementia.

**Results:**

Of 364 consecutive brain donors (100% male; 53 [14.6%] self-identified as Black and 311 [85.4%] as White; median [IQR] age, 65 [47-77] years) 20 years or older, there were 294 individuals with CTE and 70 controls. Among donors older than 65 years, *APOE*ε4 status was significantly associated with CTE stage (odds ratio [OR], 2.34 [95% CI, 1.30-4.20]; false discovery rate [FDR]–corrected *P* = .01) and quantitative p-tau burden in the dorsolateral frontal lobe (β, 1.39 [95% CI, 0.83-1.94]; FDR-corrected *P* = 2.37 × 10^−5^). There was a nonsignificant association between *APOE*ε4 status and dementia (OR, 2.64 [95% CI, 1.06-6.61]; FDR-corrected *P* = .08). Across 11 brain regions, significant associations were observed for semiquantitative p-tau burden in the frontal and parietal cortices, amygdala, and entorhinal cortex (OR range, 2.45-3.26). Among football players, the *APOE*ε4 association size for CTE stage was similar to playing more than 7 years of football. Associations were significantly larger in the older half of the sample. There was no significant association for CTE status. Association sizes were similar when donors with an Alzheimer disease neuropathological diagnosis were excluded and were reduced but remained significant after adjusting for neuritic and diffuse amyloid plaques. No associations were observed for *APOE*ε2 status. Models were adjusted for age at death and race.

**Conclusions and Relevance:**

*APOE*ε4 may confer increased risk for CTE-related neuropathological and clinical outcomes among older individuals with RHI exposure. Further work is required to validate these findings in an independent sample.

## Key Points

**Question:**

Are *APOE*ε4 and ε2 associated with chronic traumatic encephalopathy (CTE) neuropathology and related endophenotypes?

**Findings:**

In this genetic association study of 364 brain donors with repetitive head impact exposure from contact sports or military service (294 with and 70 without neuropathologically confirmed CTE), *APOE*ε4 was significantly associated with CTE stage and quantitative phosphorylated tau burden in the dorsolateral frontal lobe among those older than 65 years. The *APOE*ε4 association size for CTE stage was similar to playing more than 7 years of football; no associations were observed for *APOE*ε2.

**Meaning:**

*APOE*ε4 may confer increased risk for CTE-related neuropathological and clinical outcomes among older individuals with repetitive head impact exposure.

## Teaser

This genetic association study tests the association between *APOE* genotype and chronic traumatic encephalopathy neuropathology and related endophenotypes.

## Introduction

Chronic traumatic encephalopathy (CTE) is a distinct neurodegenerative disease associated with exposure to repetitive head impacts (RHIs).^1^ Although clinical consensus criteria were recently proposed, they have not been validated and the criterion standard diagnosis remains neuropathologic.^2^ The pathognomonic lesion in CTE is hyperphosphorylated tau located perivascularly, usually at the depths of the sulci.^3^ Most individuals diagnosed with CTE have played organized contact sports, most commonly US football and boxing, although CTE has also been described in veterans with RHI exposure.^4^ Among football players, duration of play has been most definitively linked with CTE pathology.^5^ Not all individuals who play contact sports develop CTE and there is marked variation in the extent of pathology among individuals with CTE, suggesting that risk factors beyond RHI, including genetic factors, may play a role.^6^

*APOE*ε4, which codes for the primary cholesterol transporter in the brain, confers the greatest genetic risk for sporadic Alzheimer disease (AD), while the ε2 allele confers protection.^7,8^ The ε4 allele confers varying risk for AD at different ages and ε4 carriers may show better cognitive performance in young adulthood than non-ε4 carriers.^9,10,11^ *APOE*ε4 also has been implicated as a risk factor for poor recovery after traumatic brain injury (TBI) and following exposure to contact sports.^12,13,14,15,16^ Further, *APOEε4* may moderate the association of TBI and AD.^17^ Given *APOEε4’s* link to AD and TBI, Stern et al^18^ assessed its role in CTE, finding that *ε4* homozygotes were overrepresented among 68 individuals with neuropathologically confirmed CTE without other neurodegenerative diseases, compared with the general population. Stein et al^19^ found that among 88 individuals with CTE, the *ε4* allele was associated with deposition of amyloid-β (Aβ) and that deposition of Aβ was associated with more severe CTE burden. The sample sizes in these studies were small, did not directly test the ε4 association with CTE, and/or did not include a group of RHI-exposed controls (ie, individuals without CTE). Here, we address these limitations in the largest study of the *APOE*-CTE association to date and to our knowledge. In addition to a traditional case-control analysis, we investigate the ε4 association with CTE stage and semiquantitative and quantitative regional tau measures and dementia, investigate whether the associations are independent of AD pathology, present findings stratified by age, compare the relative associations of ε4 with duration of play among football players, and investigate ε4 duration of play interactions. We repeat analyses for *APOEε2.*

## Methods

The eMethods in the Supplement provides additional methodological details. Methods followed Strengthening the Reporting of Genetic Association Studies (STREGA) reporting guideline.^20^

### Description of Donors

Donors from the Veterans Affairs–Boston University–Concussion Legacy Foundation Brain Bank^21^ were recruited between February 2008 to August 2019. To be eligible, donors needed to have a history of RHI exposure (eg, contact sports or military service), regardless of whether symptoms manifested during life. Donors were required to have RHI exposure because most individuals found to have CTE have had RHI exposure. Included in this analysis were men who self-identified as Black or White. We did not include other races because of small sample sizes and allele frequency differences across races. eFigure 1 in the Supplement shows a flowchart of included and excluded donors. Donors’ next of kin provided written consent; institutional review board approval was obtained through Boston University Medical Campus and Bedford Veterans Affairs Hospital.

### DNA Genotyping

DNA extracted from brain tissue samples was genotyped at 2 single nucleotide polyvariations, rs429358 and rs7412, using TaqMan assays (Applied Biosystems) to determine 6 possible *APOE* genotypes (ε2/ε2, ε2/ε3, ε2/ε4, ε3/ε3, ε3/ε4, ε4/ε4).

### Clinical Evaluation

Researchers conducting retrospective clinical evaluations with informants were completely blind to the neuropathological analysis. Informants were interviewed before receiving the results of the neuropathological examination. Evaluations included collection of demographics, educational attainment, athletic history, military history, TBI history, and a timeline of cognitive, behavioral, mood and motor symptomology. A dementia diagnosis using *Diagnostic and Statistical Manual of Mental Disorders* (Fourth Edition) criteria^22^ was made based on consensus among at least 2 doctoral-level clinicians (B.D., D.H.D., D.I.K., L.E.G., R.C.C., N.W.K., M.L.A., or J.M.).

### Neuropathological Evaluation

Neuropathologists, blinded to the donor’s RHI exposure and clinical history, diagnosed CTE using the National Institute of Neurological Disorders and Stroke/National Institute of Biomedical Imaging and Bioengineering neuropathological criteria.^3^ Donors diagnosed with CTE also were assigned a CTE stage (I-IV, increasing with severity) using validated criteria.^21^ Neuropathologists recorded semiquantitative measures of phosphorylated tau (p-tau) burden (by AT8 immunostaining) on a scale of 0 to 3 (with increasing severity) for 11 prespecified regions (dorsolateral frontal [DLFL], inferior orbital frontal, superior temporal, inferior parietal, hippocampus CA1, hippocampus CA2/3, hippocampus CA4, entorhinal cortex, amygdala, locus coeruleus, substantia nigra) commonly affected in CTE. Global burden of neuritic and diffuse Aβ plaques were assessed with Bielschowsky silver and Aβ (4G8 antibody clone) staining respectively on a scale of 0 to 3 (with increasing severity).

AT8-immunostained slides from the DLFL were scanned and digitized at 20× magnification using the Aperio ScanScope (Leica).^23^ We focused on the DLFL because this region is typically first affected and subsequently incurs substantial tau burden in CTE.^21,24^ Quantification was standardized to the area measured and presented as positive pixel count per mm^2^.

### Statistical Analysis

Missing values for neuropathological outcomes (semiquantitative and quantitative tau measures) were imputed using multiple imputation by chained equations. All genetic models were dominant (ie, having 1 or 2 copies of the allele was considered equivalent) rather than additive or recessive to maintain a sufficient number of carriers in each outcome group. Regression models were adjusted for self-reported race and age at death. Age at death was included as a covariate as it has been previously found to be associated with CTE stage.^1,24^ We selected the median age for age-stratified analyses to have similar power to detect an association in each group and because age 65 years is frequently used to distinguish early- from late-onset dementia.^25^ Among all donors and in median age-stratified analyses, we tested the association of *APOE*ε4 with CTE status and with dementia in separate, multivariable binary logistic regression models. Among all brain donors and in median age-stratified analyses, we tested the association of *APOE*ε4 with CTE stage (0-IV; 0 = no CTE pathology) and semiquantitative tau burden across the 11 brain regions (0-3) in separate, multivariable ordinal logistic regression models. Among all brain donors and in median age-stratified analyses, we tested the association of *APOE*ε4 with quantitative tau burden in the DLFL in linear regression models. To test whether the *APOE*ε4 association was independent of an AD Reagan neuropathological diagnosis (ie, intermediate or high likelihood of dementia due to AD) and Aβ pathology, we conducted sensitivity analyses excluding donors with an AD neuropathological diagnosis and further adjusting for measures of neuritic and diffuse plaques. To compare the relative association sizes of *APOE*ε4 with age and duration of play among football players, we repeated the above regression models among all brain donors who played football, adding a duration of play term in years. Lastly, we conducted identical analyses for *APOE*ε2 (eTable 11 in the Supplement). *P* values were 2-sided and statistical significance was set at .05 after false discovery rate (FDR) correction (4 tests for the primary outcomes: CTE, CTE stage, dementia, quantitative tau burden in the dorsal lateral frontal lobe; 11 tests for the regional semi-quantitative tau burden). Analysis took place between June 2020 and April 2022.

## Results

The study included 364 male donors, all of whom had a history of exposure to RHI from contact sports or military service. The median (IQR) age was 65.0 (47.0-77.0) years, and 53 (14.6%) were Black. The sample included 294 brain donors with neuropathologically confirmed CTE (80.8%) and 70 brain donors without evidence of CTE pathology (19.2%). Of 294 donors with CTE, 42 (14.3%) had stage I CTE, 63 (21.4%) had stage II, 96 (32.7%) had stage III, and 93 (31.6%) had stage IV. Table 1 shows demographic, RHI-related, clinical, *APOE*, and other neuropathological characteristics of donors stratified by CTE status and stage (among those with CTE). eTables 1 and 2 in the Supplement show the same characteristics stratified by median age (65 years). The overall *APOE*ε4 and ε2 allele frequencies were 0.20 (0.23 for individuals with neuropathologically confirmed CTE; 0.16 for controls) and 0.06 (0.06 for individuals with neuropathologically confirmed CTE; 0.06 for controls), respectively. *APOE* genotype frequencies did not significantly differ from Hardy Weinberg equilibrium. eTable 3 in the Supplement shows missingness for semiquantitative and quantitative tau pathology measures that were imputed in our models. eFigure 2 in the Supplement shows plots of the actual and predicted values from the imputation for each neuropathological region. Missingness across most regions was significantly associated with age of death, suggesting that missingness can be explained by other observed variables (ie, missing at random), a necessary condition for multiple imputation, and is less likely a function of the value of the variable itself (ie, missing not at random).

**Table 1.** Demographic, Clinical, Head Trauma–Related, Genetic, and Neuropathological Characteristics Stratified by CTE Control Status and CTE Stage

Characteristic	No. (%)						
	Total (N = 364)	Controls (n = 70)	CTE (n = 294)	Stage I (n = 42)	Stage II (n = 63)	Stage III (n = 96)	Stage IV (n = 93)
Demographic and clinical							
Age, median (IQR) [range], y	65.0 (47.0-77.0) [20-98]	57.0 (35.0-73.3) [20-89]	67.0 (49.0-78.0) [20-98]	34.5 (24.8-63.3) [20-89]	52.0 (34.0-67.0) [21-89]	66.0 (53.8-76.8) [25-89]	77.0 (69.0-82.5) [46-98]
Self-reported Black race	53 (14.6)	6 (8.6)	47 (16.0)	6 (14.3)	7 (11.1)	24 (25.0)	10 (10.8)
Self-reported White race	311 (85.4)	64 (91.4)	247 (84.0)	36 (85.7)	56 (88.9)	72 (75.0)	83 (89.2)
Dementia	205 (56.3)	27 (38.6)	178 (60.5)	13 (31.0)	21 (33.3)	57 (59.4)	87 (93.5)
Cognitive symptoms present	327 (89.8)	55 (78.6)	272 (92.5)	35 (83.3)	55 (87.3)	90 (93.8)	92 (98.9)
Cause of death							
Suicide	53 (14.6)	16 (22.9)	37 (12.6)	13 (31.0)	15 (23.8)	8 (8.3)	1 (1.1)
Unintentional overdose	18 (4.9)	5 (7.1)	13 (4.4)	1 (2.4)	6 (9.5)	6 (6.3)	0
Cardiovascular disease	60 (16.5)	6 (8.6)	54 (18.4)	5 (11.9)	15 (23.8)	27 (28.1)	7 (7.5)
Neurodegenerative disease	129 (35.4)	20 (28.6)	109 (37.1)	5 (11.9)	11 (17.5)	24 (25.0)	69 (74.2)
Motor neuron disease	17 (4.7)	2 (2.9)	15 (5.1)	1 (2.4)	5 (7.9)	7 (7.3)	2 (2.2)
Cancer	23 (6.3)	3 (4.3)	20 (6.8)	2 (4.8)	3 (4.8)	9 (9.4)	6 (6.5)
Injury	8 (2.2)	2 (2.9)	6 (2.0)	2 (4.8)	0	2 (2.1)	2 (2.2)
Other	54 (14.8)	15 (21.4)	39 (13.3)	13 (31.0)	7 (11.1)	13 (13.5)	6 (6.5)
Unknown	1 (0.3)	1 (1.4)	0	0	0	0	0
Head trauma–related							
Contact sports	353 (97.0)	59 (84.3)	294 (100.0)	42 (100.0)	63 (100.0)	96 (100.0)	93 (100.0)
Age of first exposure to contact sports, mean (SD) [range], y	11.8 (3.5) [3-34]	11.6 (4.5) [4-34]	11.8 (3.3) [3-25]	10.1 (3.9) [3-16]	11.4 (3.7) [3-25]	12.0 (2.8) [5-20]	12.7 (2.7) [5-20]
Football	323 (88.7)	52 (74.3)	271 (92.2)	36 (85.7)	54 (85.7)	95 (99.0)	86 (92.5)
Professional	181 (49.7)	12 (17.1)	169 (57.5)	10 (23.8)	25 (39.7)	65 (67.7)	58 (62.4)
College/semiprofessional	113 (31.0)	14 (20.0)	99 (33.7)	14 (33.3)	23 (36.5)	27 (28.1)	27 (29.0)
High school/youth	52 (14.3)	26 (37.1)	26 (8.8)	12 (28.6)	6 (9.5)	3 (3.1)	1 (1.1)
Duration of football play, mean (SD) [range], y	12.9 (5.9) [1-33]	7.9 (4.6) [1-21]	13.9 (5.6 [1-33]	10.0 (4.9) [1-20]	12.4 (4.7) [3-25]	14.9 (5.4) [1-27]	15.2 (5.8) [4-33]
Hockey	13 (3.6)	2 (2.9)	11 (3.7)	4 (9.5)	5 (7.9)	1 (1.0)	1 (1.1)
Soccer	6 (1.6)	2 (2.9)	4 (1.4)	2 (4.8)	2 (3.2)	0	0
Amateur wrestling	2 (0.5)	2 (2.9)	0	0	0	0	0
Boxing	6 (1.6)	0	6 (2.0)	1 (2.4)	0	0	5 (5.4)
Rugby	4 (1.1)	0	4 (1.4)	0	2 (3.2)	0	2 (2.1)
Other contact sports	4 (1.1)	1 (1.4)	3 (1.0)	2 (4.8)	1 (1.6)	0	0
Military	97 (26.6)	21 (30.0)	76 (25.9)	10 (23.8)	8 (12.7)	19 (19.8)	39 (41.9)
Combat	20 (5.5)	8 (11.4)	12 (4.1)	4 (9.5)	3 (4.8)	0	5 (5.4)
APOE							
ε2 Carriers	42 (11.5)	8 (11.4)	34 (11.6)	4 (9.5)	7 (11.1)	10 (10.4)	13 (14.0)
ε4 Carriers	128 (35.2)	20 (28.6)	108 (36.7)	12 (28.6)	18 (28.6)	32 (33.3)	46 (49.5)
ε2ε2	3 (0.8)	1 (1.4)	2 (0.7)	0	1 (1.6)	0	1 (1.1)
ε2ε3	29 (8.0)	5 (7.1)	24 (8.2)	4 (9.5)	5 (7.9)	9 (9.4)	6 (6.5)
ε2ε4	10 (2.7)	2 (2.9)	8 (2.7)	0	1 (1.6)	1 (1.0)	6 (6.5)
ε3ε3	204 (56.0)	44 (62.9)	160 (54.4)	26 (61.9)	39 (61.9)	55 (57.3)	40 (43.0)
ε3ε4	102 (28.0)	15 (21.4)	87 (29.6)	10 (23.8)	17 (27.0)	27 (28.1)	33 (35.5)
ε4ε4	16 (4.4)	3 (4.3)	13 (4.4)	2 (4.8)	0	4 (4.2)	7 (7.5)
Pathology							
Log quantitative tau burden in dorsolateral frontal lobe, mean (SD) [range], tau + pixels/mm^2^	7.3 (2.2) [3.3-13.3]	5.9 (1.7) [3.5-10.8]	7.6 (2.2) [3.3-13.3]	5.3 (0.9) [3.3-7.6]	6.2 (1.7) [3.6-12.6]	7.4 (1.4) [4.6-11.4]	9.6 (1.6) [6.3-13.3]
AD pathology	55 (10.6)	12 (17.1)	43 (14.6)	2 (4.8)	3 (4.8)	8 (8.3)	30 (32.3)
CERAD neuritic plaque score, mean (SD)	0.61 (1.00)	0.48 (0.95)	0.63 (1.0)	0.15 (0.43)	0.40 (1.28)	0.45 (0.79)	1.22 (0.97)
Braak NFT stage, mean (SD)	2.39 (2.06)	1.58 (2.25)	2.58 (1.96)	0.75 (1.28)	1.37 (1.47)	2.92 (1.58)	3.99 (1.65)
Lewy body pathology	63 (12.2)	11 (15.7)	52 (17.7)	5 (11.9)	5 (7.9)	17 (17.7)	25 (26.9)
Brainstem predominant	37 (7.1)	4 (5.7)	33 (11.2)	3 (7.1)	3 (4.8)	8 (8.3)	19 (20.4)
Limbic/neocortical predominant	26 (5.0)	7 (10.0)	19 (6.5)	2 (4.8)	2 (3.2)	9 (9.4)	6 (6.5)
FTLD tau	22 (6.0)	6 (8.6)	16 (5.4)	2 (4.8)	3 (4.8)	5 (5.2)	6 (6.5)
FTLD tdp-43	19 (5.2)	2 (2.9)	17 (5.8)	2 (4.8)	3 (4.8)	1 (1.0)	11 (11.8)

Abbreviations: AD, Alzheimer disease; CERAD, Consortium to Establish a Registry for Alzheimer Disease; CTE, chronic traumatic encephalopathy; FTLD, frontotemporal lobar degeneration; NFT, neurofibrillary tangle.

Among the full sample, there were significant *APOE*ε4–age group interactions for outcomes CTE stage, quantitative tau burden in the DLFL, and dementia (Table 2) and therefore results are presented stratified by median age. As shown in Table 2, in the older group, *APOE*ε4 status was significantly associated with CTE stage, increasing the odds of increasing one level by 2.34 (95% CI, 1.30-4.20; FDR-corrected *P* = .01). The test of parallel lines was nonsignificant, suggesting the proportional odds assumption holds. *APOE*ε4 status was significantly associated with quantitative tau burden in the DLFL, increasing the log transformed AT8+ pixel count per mm^2^ by 1.39 units (95% CI, 0.83-1.94; FDR-corrected *P* = 2.37 × 10^−5^). The data suggest that there may be an association between *APOE*ε4 status and dementia, but this was not significant (odds ratio, 2.64 [95% CI, 1.06-6.61]; FDR-corrected *P* = .08). There was no significant association between *APOE*ε4 status and CTE status. In the younger group, there were no significant associations. In sensitivity analyses excluding self-reported Black donors, associations were in consistent directions with somewhat varying sizes (eTable 4 in the Supplement), particularly for dementia. In sensitivity analyses, using different age cut points for age-stratified analyses, association sizes increased with increasing age of stratification for the older group (eTable 5 in the Supplement). There were no significant associations for the younger group for any of the age cut points.

**Table 2.** Estimated Associations of *APOE*ε4 Status With CTE Diagnosis, CTE Stage, Dementia Diagnosis, and Quantitative Tau Burden in the Dorsolateral Frontal Lobe^a^

Outcome	Age ≤65 y (n = 183)		Age >65 y (n = 181)		Age >65 y, excluding donors meeting AD Reagan criteria (n = 136)	
	OR (95% CI)	FDR-corrected *P* value	OR (95% CI)	FDR-corrected *P* value	OR (95% CI)	FDR-corrected *P* value
CTE diagnosis	1.30 (0.61-2.80)	.54	1.30 (0.53-3.22)	0.57	1.66 (0.65-6.39)	.54
CTE stage^b^	1.23 (0.70-2.17)	.54	2.34 (1.30-4.20)	0.01	3.13 (1.66-6.39)	.01
Dementia	0.67 (0.30-1.52)	.51	2.64 (1.06-6.61)	0.08	2.37 (1.02-6.56)	.17
Quantitative tau burden in dorsolateral frontal lobe, β (95% CI)^c^	0.59 (0.04-1.14)^d^	.08	1.39 (0.83-1.94)^d^	2.37 × 10^−5^	1.04 (0.48-1.60)^d^	2.29 × 10^−3^

Abbreviations: AD, Alzheimer disease; CTE, chronic traumatic encephalopathy; OR, odds ratio.

^a^ All analyses are adjusted for age and self-reported race. In models including the full sample, the *P* values for the age group–*APOE* ε4 interaction were .05, .03, and .06 for outcomes CTE stage, dementia, and quantitative tau burden in dorsolateral frontal lobe, respectively.

^b^ ORs are the odds of increasing 1 stage (scale of 0-4) for ε4 carriers compared with noncarriers.

^c^ Beta value is the increase in log tau + pixels/mm^2^ in the dorsolateral frontal lobe for ε4 carriers compared with noncarriers.

^d^ β (95% CI) is reported.

**Figure 1.**
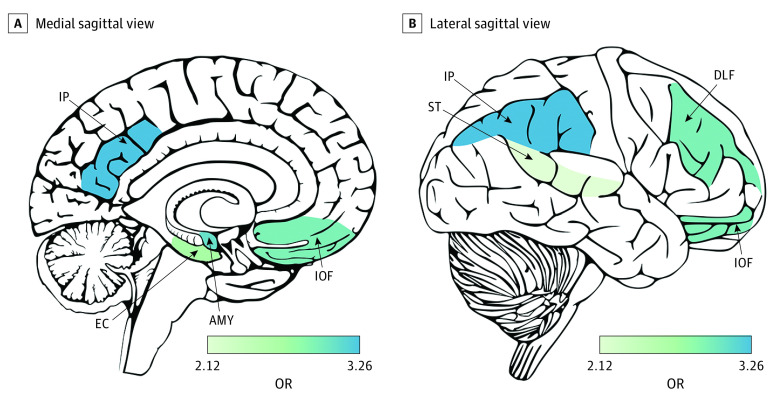
Brain Heat Map of Estimated Associations of *APOE*ε4 Status With Semiquantitative Tau Burden in Brain Regions Commonly Affected in Chronic Traumatic Encephalopathy Among Donors Older Than 65 Years Only regions with at least nominally significant associations shown. Odds ratio (OR) is the odds of increasing 1 level (scale of 0-3) for *APOE*ε4 carriers compared with noncarriers. Generated with cerebroViz.^26^ AMY indicates amygdala; DLF, dorsal lateral frontal; EC, entorhinal cortex; IOF, inferior orbital frontal; IP, inferior parietal; ST, superior temporal.

In models testing the association between *APOE*ε4 status and tau burden in regions commonly affected in CTE, significant associations were observed in the older age group in the frontal and parietal cortex, amygdala, and entorhinal cortex (odds ratio range, 2.45-3.26). In the younger group, association sizes were in the same direction but were markedly smaller and were not significant after correction for multiple testing (Figure 1 and eTable 5 in the Supplement).^26^

**Figure 2.**
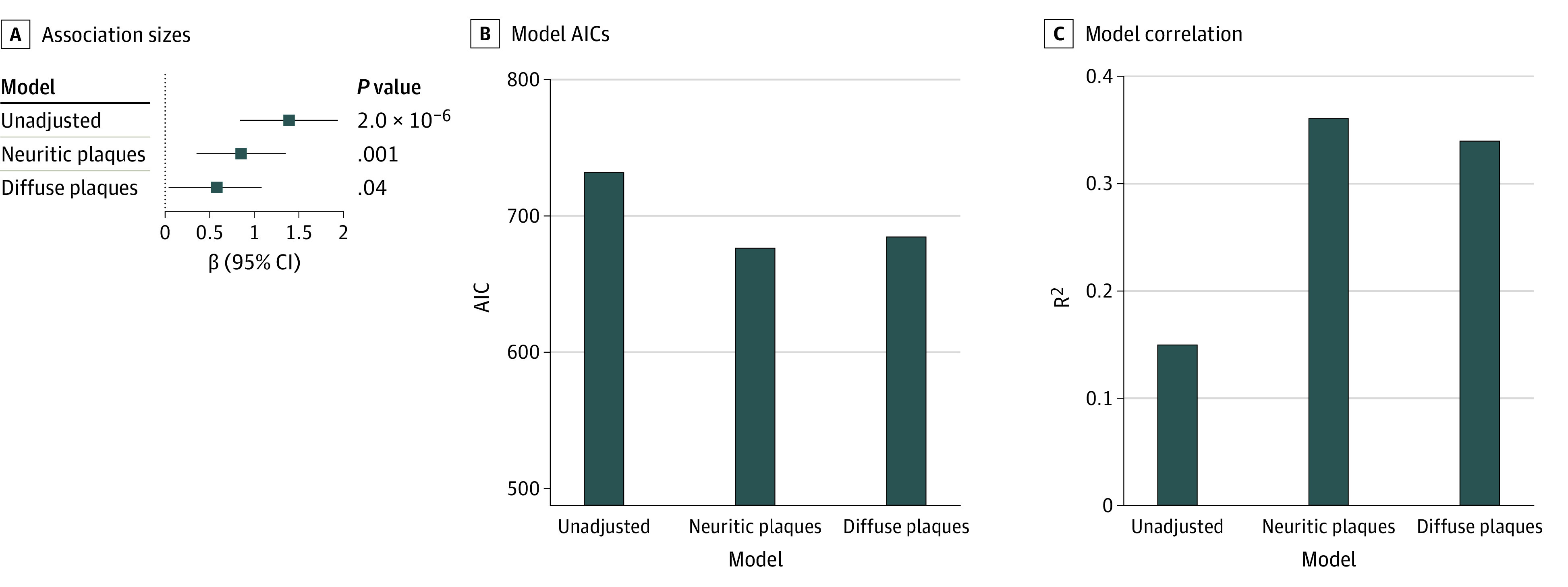
Estimated Associations of *APOE*ε4 Status With Quantitative Tau Burden in the Dorsolateral Frontal Lobe When Adjusting for Amyloid-β Pathology A, Association sizes unadjusted and adjusted for neuritic and diffuse amyloid plaques. β Value is the increase in log tau + pixels/mm^2^ in the dorsolateral frontal lobe for ε4 carriers compared with noncarriers. The whiskers represent 95% CIs. B, Akaike information criterion (AIC) for unadjusted and adjusted models. Smaller AIC indicates better model fit. C, Variance explained (*r^2^*) for unadjusted and adjusted models.

In sensitivity analyses of excluded donors who met Reagan criteria for an AD neuropathological diagnosis, association sizes were larger for CTE stage, similar for dementia, and modestly reduced for measures of regional tau burden (Table 2, Figure 1, eTable 5 in the Supplement). Figure 2 and eTable 6 in the Supplement show sensitivity analyses among the older group for the model with quantitative tau burden in the DLFL as the outcome in which we additionally adjusted for neuritic and diffuse Aβ plaque pathology. The addition of neuritic and diffuse Aβ plaque pathology as covariates resulted in reductions in *APOE*ε4 association sizes, increases in variance of the outcome explained (*r^2^*), and better model fit (Akaike information criterion). Reduction in *APOE*ε4 association size was greater for diffuse plaques (58%) than neuritic plaques (39%), and *r^2^* and Akaike information criterion were similar for models with adjustment for neuritic and diffuse plaques. Associations remained significant after adjustment, suggesting some of the association was independent of Aβ pathology.

In models limited to football players, we compared association sizes of *APOE*ε4 with duration of play in years. Unlike duration of play, which had similar association sizes in both age groups, *APOE*ε4 association sizes differed markedly by age group. Among the older football players, for CTE stage as the outcome, the association size for *APOE*ε4 status was similar to playing more than 7 years of football (Table 3). For the primary outcomes, in age-stratified analyses, we observed one significant association for the interaction between *APOE*ε4 status and duration of play in years on odds of dementia in the young group (odds ratio, 0.78 [95% CI, 0.66-0.91]; FDR-corrected *P* = .01) (eTables 7 and 8 in the Supplement). eTables 9 and 10 in the Supplement show analyses repeated for *APOE*ε2. We did not observe any significant associations.

**Table 3.** Estimated Associations of *APOE*ε4 Status, Age at Death, and Duration of Play With CTE Status, CTE Stage, and Quantitative Tau Burden in the Dorsolateral Frontal Lobe in US Football–Playing Donors^a^

Outcome	Age ≤65 y (n = 160)		Age >65 y (n = 163)		Age >65 y, excluding donors meeting AD Reagan criteria (n = 123)	
	OR (95% CI)	FDR-corrected *P* value	OR (95% CI)	FDR-corrected *P* value	OR (95% CI)	FDR-corrected *P* value
CTE status						
APOEε4	0.81 (0.32 to 2.07)	.71	1.31 (0.38 to 4.45)	.71	1.07 (0.21 to 5.36)	.93
Age, y	1.02 (0.99 to 1.05)	.23	1.05 (0.96 to 1.14)	.38	1.08 (0.96 to 1.21)	.26
Duration of play, y	1.25 (1.13 to 1.38)	1.98 × 10^−4^	1.28 (1.13 to 1.45)	5.61 × 10^−4^	1.32 (1.13 to 1.55)	2.36 × 10^−3^
CTE stage (0-IV; ordinal)						
APOEε4	0.88 (0.47 to 1.66)	.72	3.35 (1.70 to 6.61)	1.88 × 10^−3^	3.64 (1.61 to 8.24)	6.27 × 10^−3^
Age, y	1.05 (1.03 to 1.07)	7.47 × 10^−5^	1.07 (1.02 to 1.12)	9.20 × 10^−3^	1.10 (1.04 to 1.17)	1.88 × 10^−3^
Duration of play, y	1.18 (1.11 to 1.25)	6.90 × 10^−6^	1.17 (1.11 to 1.25)	5.54 × 10^−6^	1.19 (1.11 to 1.28)	1.63 × 10^−5^
Dementia						
APOEε4	0.80 (0.34 to 1.85)	.67	2.54 (0.94 to 6.85)	.11	1.97 (0.69 to 5.60)	.27
Age, y	1.12 (1.07 to 1.16)	3.78 × 10^−6^	1.10 (1.03 to 1.18)	.02	1.12 (1.04 to 1.20)	9.20 × 10^−3^
Duration of play, y	1.02 (0.95 to 1.10)	.67	0.98 (0.91 to 1.05)	.67	0.97 (0.90 to 1.05)	.56
Quantitative tau burden in dorsolateral frontal lobe (continuous)						
APOEε4	0.47 (−0.12 to 1.05)^b^	.18	1.40 (0.82 to 1.97)^b^	2.35 × 10^−5^	1.08 (0.51 to 1.66)^b^	1.17 × 10^−3^
Age, y	0.05 (0.04 to 0.07)^b^	2.96 × 10^−6^	0.04 (−0.005 to 0.08)^b^	.13	0.07 (0.02 to 0.11)^b^	6.96 × 10^−3^
Duration of play, y	0.06 (0.01 to 0.12)^b^	.03	0.06 (0.01 to 1.11)^b^	.03	0.05 (0.01 to 0.10)^b^	.04

Abbreviations: AD, Alzheimer disease; CTE, chronic traumatic encephalopathy; FDR, false discovery rate; OR, odds ratio.

^a^ All analyses are also adjusted for race.

^b^ β (95% CI) is reported.

## Discussion

We examined the association between *APOE* and CTE neuropathological endophenotypes among 294 individuals with neuropathologically confirmed CTE and 70 brain donors without evidence of CTE pathology, all with RHI exposure from contact sports or military service. Among donors older than 65 years, we observed significant associations for the associations of *APOE*ε4 status with CTE stage and quantitative and semiquantitative measures of tau pathology. Associations were strongest in the cortex and remained significant, albeit attenuated when models were adjusted for Aβ pathology. Associations were large, albeit nonsignificant after FDR correction, for dementia. Associations were reduced and nonsignificant among donors 65 years or younger. In analyses limited to former football players older than 65 years, the association size of *APOE*ε4 status with CTE stage was similar to playing more than 7 years of football. We did not observe any associations for *APOE*ε4 status with CTE status or for *APOE*ε2 status on any outcome.

*APOE* is the most investigated gene regarding outcomes following TBI. Several meta-analyses suggest that the ε4 allele confers a small risk of poor outcomes following TBI, including functional outcome measures and neuropsychological performance months to years after the event.^14,15,16^ *APOE*ε4 has been speculated to lead to worse outcomes after TBI via several mechanisms including direct neurotoxicity, modulation of tau biology, abnormal cerebrovascular function, effects on the blood-brain barrier, inflammation, and oxidant injury.^27,28,29,30,31,32^ Several of these same mechanisms have been suggested and/or implicated as catalysts of CTE pathogenesis. For example, impaired neurovascular unit function and loss of blood-brain barrier integrity have been observed in postmortem brains of athletes with CTE.^33^ Additionally, in contact sport athletes with neuropathologically confirmed CTE, elevated CD68-reactive microglia staining in the frontal cortex, a marker of neuroinflammation, has been shown to correlate with CTE disease severity.^23^ *APOE*ε4 carriers may experience greater secondary injury and impaired capacity for recovery from these processes induced by TBI.^31^

*APOE*ε4 is the strongest genetic risk factor for sporadic AD, incurring a risk of 2 to 3 times for 1 copy and as much as 14 times for 2 copies among individuals of European ancestry.^7^ When we excluded donors who met Reagan criteria for an AD neuropathological diagnosis, association sizes only changed modestly. Our findings are in line with recent work showing an association of *APOE*ε4 with tau burden as measured by [^18^F]-AV-1451 tracer signal in the cortical gray matter in 34 former contact sport athletes unlikely to have AD based on their biomarker profile.^34^

Traditionally in AD, the effect of *APOE*ε4 has been thought to be mediated through Aβ pathology. ApoE4 is known to impair Aβ clearance and accelerate Aβ synthesis and fibril formation and deposition.^35,36^ Aβ deposition triggers downstream hyperphosphorylation and aggregation of tau protein in neurofibrillary tangles. However, more recent evidence suggests that *APOE*ε4 may induce tau seeding via an Aβ-independent mechanism.^37^ Indeed, we found that although the *APOE*ε4 association sizes were reduced after adjusting for Aβ pathology, there was also an Aβ-independent association. Given the inherent cross-sectional nature of brain bank studies, we were not able to discern the temporal relationships of these pathologies or to do a formal mediation analysis. Nonetheless, we do note that both the highest frequencies of *APOE*ε4 carriers and AD pathology were among donors with CTE stage IV pathology.

*APOE*ε4 associations were significantly larger among donors older than 65 years compared with donors 65 years and younger. This was in contrast with age at death and duration of play, which showed similar association sizes in both age groups. Interestingly, in post hoc sensitivity analyses among the older group, *APOE*ε4 association sizes increased with increasing age of stratification, suggesting risk may continue to increase with increasing age, at least up to age 75 years. The relationships between age, *APOE*ε4, and neurological outcomes are complex. *APOE*ε4 carriers have an earlier age of AD symptom onset, and a similar relationship was recently shown for frontotemporal dementia.^38,39^ It has also been shown that the largest *APOE*ε4 association with AD incidence is among individuals aged 65 to 70 years, with smaller associations in those younger than 55 years and older than 85 years.^9^ There is also literature on the protective associations of *APOE*ε4 with cognition in younger and middle-aged healthy adults.^10,11^ For instance, a recent study that examined *APOE*ε4 association with cognition across the lifespan found ε4 heterozygotes had better performance between age 45 and 55 years, and worse performance in individuals older than 75 years.^40^ The antagonistic pleiotropy hypothesis postulates that a gene may have varying effects on health outcomes during different life stages, and this may explain the age-varying associations of *APOE*ε4, including with CTE.^41^

In people with AD, having *APOE*ε4 is associated with increased burden of tau pathology in medial temporal structures, relative to the cortex.^42,43^ In our sample greatly enriched for CTE, the strongest associations of *APOE*ε4 with tau pathological burden were in the cortex, as well as the amygdala and entorhinal cortex, but not the hippocampus. The cortical regions and the entorhinal cortex are affected early in CTE (stages I and II), while the amygdala is affected later in the disease course (stages III and IV). Our findings suggest *APOE*ε4 likely has associations both with early and late pathology of CTE. We have shown previously that late-stage CTE clusters into a group with predominant cortical p-tau pathology and a group with predominant medial temporal p-tau pathology.^24^ Future work disentangling how age, RHI exposure, and genetic background together impact the relative distribution of tau pathology and clinical syndrome in CTE may provide important insights for understanding disease mechanisms, course, and therapies. Similarly, *APOE*ε4 is a well-established risk factor for cardiovascular disease, which commonly affects former elite football players.^44,45^ How cardiovascular disease may mediate or moderate the associations of *APOE*ε4 with CTE-related outcomes will be important to investigate.

Among football players, comparison of the size of associations for *APOE*ε4 and duration of play with the various outcomes may provide additional insight into CTE initiation and progression. As we have shown previously, duration of play was strongly associated with CTE status and with CTE stage, which is defined by the location of p-tau pathology, and to a lesser extent burden of p-tau pathology.^1^ Duration of play was weakly or not associated with quantitative tau burden in the DLFL and dementia. Conversely, *APOE*ε4 status was not associated with CTE status but showed a robust association with quantitative tau burden in the DLFL and a large association size with dementia (albeit nonsignificant after FDR correction). Dementia may be predominantly driven by burden of tau pathology in the cortex, as has been shown in AD.^46^ Taken together, these findings suggest that duration of play may drive disease initiation and have a role in disease progression, while *APOE*ε4 may be particularly important for disease progression and severity. Along with the finding that ε4 status may confer similar risk on CTE stage as playing more than 7 years of football, these insights suggest a first step toward a precision medicine approach to harm reduction and interindividual risk. They also provide insights into necessary/sufficient components of a causal pie in CTE. Additional work with larger sample sizes and in prospectively assessed samples will be needed to better understand these relationships.

*APOE*ε2, the least common of the 3 *APOE* alleles, has a protective effect for AD and risk and protective effects for several other neurological diseases including stroke, cerebral amyloid angiopathy, posttraumatic stress disorder, age-related macular degeneration, progressive supranuclear palsy, and argyrophilic grain disease.^8^ Because *APOE*ε2 is less common, we had less power to detect an association. Nonetheless, for most of our association tests with the ε2 allele, association sizes were null, suggesting that even if we were better powered, we may not have identified a significant association.

### Strengths and Limitations

Strengths of this study include the largest sample size to date investigating the association between *APOE*, CTE, and its endophenotypes. Brain donors were carefully characterized, further increasing power to detect genetic associations. Additionally, all donors, both individuals with CTE and controls, had RHI exposure, putting them at risk for CTE.

The study also has limitations. Although we made use of the largest sample of brain donors with CTE in the world, the sample was still small by genetic standards, particularly after age stratification. Given the sample size, we did not model *APOE*ε4 additively, although it has been shown to have an additive association in AD. Our sample included a multiethnic sample with both self-identifying Black and White donors. Although we did adjust for self-reported race in our models, we did not adjust for population substructure because we did not have genome-wide data or ancestry informative markers on all donors. Efforts are currently underway to obtain genome-wide genotyping on all donors. We also conducted sensitivity analyses excluding self-identifying Black donors that demonstrated similar association sizes for neuropathological outcomes and more varied association sizes for dementia. We chose not to report findings among self-identifying Black donors alone because the sample size was too small to draw reliable inferences. We also did not include women in our analyses because there were too few women available in the brain bank to make meaningful inferences. Brain donors were not followed up during life and clinical and RHI exposure information came largely from retrospective informant report making recall bias a possibility.

Contact sport play has changed over time, and these changes were not reflected in our risk modeling and may have impacted the differing *APOE*ε4 associations by age. *APOE*ε4 is a well-established risk factor for shortened survival and *APOE*ε4 status, CTE pathology, and dementia may be independently associated with brain bank selection, introducing potential selection bias. Further, this selection effect is well-recognized as operating differentially in Black vs White individuals, potentially introducing additional selection pressure.^47^ The age-stratified analyses may have helped to combat potential selection bias as both *APOE*-associated survival and CTE-associated cognitive impairment are also associated with age. Larger sample sizes and increased information about the general population of individuals exposed to RHI will be needed to better understand the role of selection bias.

## Conclusions

This study provides the most concrete evidence to date that *APOE*ε4 is a risk factor for CTE-related pathological and clinical outcomes. Understanding genetic underpinnings of CTE pathology may provide insights into disease mechanism and offers a precision medicine approach to harm reduction, including guiding decisions regarding contact sport play and providing a target for therapies.

## Notes

Supplement. eMethods. eFigure 1. Flow chart of included and excluded brain donors eFigure 2. Actual and predicted values from multiple imputation for each neuropathological region eTable 1. Missingness of semi-quantitative and quantitative tau burden data across brain regions eTable 2. Demographic, clinical, head trauma-related, genetic and neuropathological characteristics stratified by CTE control status and CTE Stage among donors age>65 years eTable 3. Demographic, clinical, head trauma-related, genetic and neuropathological characteristics stratified by CTE control status and CTE Stage among donors age≤65 years eTable 4. Estimated associations of APOE ε4 status on CTE diagnosis, CTE stage, dementia diagnosis, and quantitative tau burden in the dorsolateral frontal lobe among self-reported White race, stratified by age 65 and excluding donors meeting AD Reagan criteria eTable 5. Estimated associations of APOE ε4 status on CTE diagnosis, CTE stage, dementia diagnosis, and quantitative tau burden in the dorsolateral frontal lobe, among the older age group, using different age-cut points eTable 6. Estimated associations of APOE ε4 status on semi-quantitative tau burden in brain regions commonly affected in CTE, stratified by age 65 and excluding donors meeting AD Reagan criteria eTable 7. Estimated associations of APOE ε4 status when adjusting for Aβ pathology among donors older than 65 years, with and without exclusion of donors meeting AD Reagan criteria eTable 8. Estimated associations of APOE ε4 status, years of football played and their interaction; Age>65 eTable 9. Estimated associations of APOE ε4 status, years of football played and their interaction; Age≤65 eTable 10. Estimated associations of APOE ε2 status on CTE diagnosis, CTE stage, dementia diagnosis, and quantitative tau burden in the dorsolateral frontal lobe stratified by median age 65 and overall eTable 11. Estimated associations of APOE ε2 status on semi-quantitative tau burden in brain regions commonly affected in CTE, stratified by median age 65 and overall eReferences
