# Supplementary material for: Association of APOE Genotypes and Chronic Traumatic Encephalopathy
Source: JAMA Neurol. 2022 Jun 27;79(8):787–96. doi: 10.1001/jamaneurol.2022.1634 (PMC9237800; doi:10.1001/jamaneurol.2022.1634)
Supplement: Supplement. — eMethods. eFigure 1. Flow chart of included and excluded brain donors eFigure 2. Actual and predicted values from multiple imputation for each neuropathological region eTable 1. Missingness of semi-quantitative and quantitative tau burden data across brain regions eTable 2. Demographic, clinical, head trauma-related, genetic and neuropathological characteristics stratified by CTE control status and CTE Stage among donors age>65 years eTable 3. Demographic, clinical, head trauma-related, genetic and neuropathological characteristics stratified by CTE control status and CTE Stage among donors age≤65 years eTable 4. Estimated associations of APOE ε4 status on CTE diagnosis, CTE stage, dementia diagnosis, and quantitative tau burden in the dorsolateral frontal lobe among self-reported White race, stratified by age 65 and excluding donors meeting AD Reagan criteria eTable 5. Estimated associations of APOE ε4 status on CTE diagnosis, CTE stage, dementia diagnosis, and quantitative tau burden in the dorsolateral frontal lobe, among the older age group, using different age-cut points eTable 6. Estimated associations of APOE ε4 status on semi-quantitative tau burden in brain regions commonly affected in CTE, stratified by age 65 and excluding donors meeting AD Reagan criteria eTable 7. Estimated associations of APOE ε4 status when adjusting for Aβ pathology among donors older than 65 years, with and without exclusion of donors meeting AD Reagan criteria eTable 8. Estimated associations of APOE ε4 status, years of football played and their interaction; Age>65 eTable 9. Estimated associations of APOE ε4 status, years of football played and their interaction; Age≤65 eTable 10. Estimated associations of APOE ε2 status on CTE diagnosis, CTE stage, dementia diagnosis, and quantitative tau burden in the dorsolateral frontal lobe stratified by median age 65 and overall eTable 11. Estimated associations of APOE ε2 status on semi-quantitative tau burden in brain regions commonly affec [file jamaneurol-e221634-s001.pdf]

Supplemental Online Content

Atherton K, Han X, Chung J, et al. Association of *APOE* genotypes and chronic traumatic encephalopathy. *JAMA Neurol*. Published online June 27, 2022.
doi:10.1001/jamaneurol.2022.1634

eMethods.

eFigure 1. Flow chart of included and excluded brain donors

eFigure 2. Actual and predicted values from multiple imputation for each neuropathological region

eTable 1. Missingness of semi-quantitative and quantitative tau burden data across brain regions

eTable 2. Demographic, clinical, head trauma-related, genetic and neuropathological characteristics stratified by CTE control status and CTE Stage among donors age>65 years

eTable 3. Demographic, clinical, head trauma-related, genetic and neuropathological characteristics stratified by CTE control status and CTE Stage among donors age≤65 years

eTable 4. Estimated associations of APOE ε4 status on CTE diagnosis, CTE stage, dementia diagnosis, and quantitative tau burden in the dorsolateral frontal lobe among self-reported White race, stratified by age 65 and excluding donors meeting AD Reagan criteria

eTable 5. Estimated associations of APOE ε4 status on CTE diagnosis, CTE stage, dementia diagnosis, and quantitative tau burden in the dorsolateral frontal lobe, among the older age group, using different age-cut points

eTable 6. Estimated associations of APOE ε4 status on semi-quantitative tau burden in brain regions commonly affected in CTE, stratified by age 65 and excluding donors meeting AD Reagan criteria

eTable 7. Estimated associations of APOE ε4 status when adjusting for Aβ pathology among donors older than 65 years, with and without exclusion of donors meeting AD Reagan criteria

eTable 8. Estimated associations of APOE ε4 status, years of football played and their interaction; Age>65

eTable 9. Estimated associations of APOE ε4 status, years of football played and their interaction; Age≤65

eTable 10. Estimated associations of APOE ε2 status on CTE diagnosis, CTE stage, dementia diagnosis, and quantitative tau burden in the dorsolateral frontal lobe stratified by median age 65 and overall

© 2022 Atherton K et al. *JAMA Neurology.*

eTable 11. Estimated associations of APOE ε2 status on semi-quantitative tau burden in brain regions commonly affected in CTE, stratified by median age 65 and overall

eReferences

This supplemental material has been provided by the authors to give readers additional information about their work.

© 2022 Atherton K et al. *JAMA Neurology.*

eMethods

Methods followed STrengthening the REporting of Genetic Association Studies (STREGA) guidelines.[1]

Description of Donors

Donors from the Veterans Affairs (VA)-Boston University (BU)-Concussion Legacy Foundation (CLF) Brain Bank were recruited between 2008 and 2019. To be eligible, donors needed to have a history of RHI exposure (e.g., contact sports or military service), regardless of whether symptoms manifested during life. Donors were required to have RHI exposure because most individuals found to have CTE have had RHI exposure. Included in this analysis were men who were self-identified Black and White race. Neuropathological evidence of CTE was required to be considered a case. **eFigure1** shows a flow chart of included and excluded donors. Donors' next-of-kin provided written consent for brain donation. IRB approval was obtained through Boston University Medical Campus and Bedford VA Hospital.

DNA Extraction and Genotyping

DNA was extracted from brain tissue samples using a Qiagen QIAamp DNA extraction kit (Qiagen, Valencia, CA, USA). Two single nucleotide polymorphisms, rs429358 and rs7412, were examined using TaqMan assays (Applied Biosystems, Foster City, CA, USA). to determine 6 possible *APOE* genotypes (ε2/ε2, ε2/ε3, ε2/ε4, ε3/ε3, ε3/ε4, ε4/ε4).

Clinical Evaluation

Retrospective clinical evaluations were performed using online questionnaires and structured and semi-structured post-mortem telephone interviews between researchers and informants. Researchers conducting these evaluations were completely blind to the neuropathological analysis and informants were interviewed before receiving the results of the neuropathological examination. Evaluations included collection of demographics, educational attainment, athletic

© 2022 Atherton K et al. *JAMA Neurology.*

history (type of sports played, level, position, and duration), military history (branch, location of service and duration of combat exposure), and traumatic brain injury (TBI) history (including number of concussions). A dementia diagnosis using DSM-IV criteria[2] was made based on consensus among at least two doctoral level clinicians who reviewed the medical history, including a timeline of cognitive, behavioral, mood and motor symptomology.

Neuropathological Evaluation

Neuropathological processing and gross and microscopic examination followed previously established methods.[3–6] Neuropathologists, blinded to the donor's RHI exposure and clinical history, diagnosed CTE using the NINDS/NIBIB neuropathological criteria.[7] Donors diagnosed with CTE also were assigned a CTE stage (I-IV, increasing with severity) using validated criteria.[8] Neuropathologists recorded semi-quantitative measures of phosphorylated tau burden (by AT8 immunostaining) on a 0-to-3 scale (with increasing severity) for 11 pre-specified regions (dorsolateral frontal, inferior orbital frontal, superior temporal, inferior parietal, hippocampus CA1, hippocampus CA2/3, hippocampus CA4, entorhinal cortex, amygdala, locus coeruleus, substantia nigra) commonly affected in CTE. Well-established neuropathological criteria were used to diagnose all comorbid neurodegenerative diseases.[9–24] Intermediate or high NIA Reagan criteria were used to make a neuropathological diagnosis of AD rather than the newer NIA-AA Criteria because we were not recording Thal phase prior to 2012 when the NIA-AA criteria were recommended. Additionally, cases of CTE commonly show diffuse plaques without neuritic plaques and neuritic plaques are the clearest way to differentiate AD from CTE. Global burden of neuritic and diffuse Aβ plaques were assessed with Bielschowsky silver and Aβ (4G8 antibody clone) staining respectively on a 0-3 scale (with increasing severity). AT8-immunostained slides from the dorsolateral frontal cortex were scanned and digitized at 20X magnification using the Aperio ScanScope (Leica) as previously described.[25] We focused on the dorsolateral frontal cortex because this region is affected very early and subsequently incurs

© 2022 Atherton K et al. *JAMA Neurology.*

substantial tau burden in CTE.[8,26] Briefly, the crest of the cortical sulcus (defined as the top third of two connecting gyri) was selected and highlighted in ImageScope (Lecia). The White matter/gray matter boundary was used as the outer edge of the region of interest so only gray matter was highlighted. Using Leica image analysis and automated counting software, the Aperio positive pixel count (Version 9) algorithm was used to determine the area of immunoreactivity. Quantification was standardized to the area measured and presented as positive pixel count per mm^2.

Statistical Analysis

Missing values for neuropathological outcomes (semi-quantitative and quantitative tau measures) were imputed using multiple imputation by chained equations (MICE), creating 10 imputed datasets. Age at death, race and the non-missing semi-quantitative and quantitative tau measures informed imputation. Individual imputation models used ordinal logistic regression for ordered categorical variables and predictive mean matching with 5 nearest neighbors for continuous variables. All genetic models were dominant (i.e., having 1 or 2 copies of the allele was considered equivalent) rather than additive or recessive to maintain a sufficient number of carriers in each outcome group. Regression models were adjusted for self-reported race and age at death. Age at death was included as a covariate as it has been previously found to be associated with CTE stage. [26,27,] We selected the median age for age-stratified analyses to have similar power to detect an association in each group and because age 65 is frequently used to distinguish early from late onset dementia.[28] Among all donors and in median age-stratified analyses, we tested the association of *APOE*ε4 with CTE status and with dementia in separate, multi-variable binary logistic regression models. Among all brain donors and in median age-stratified analyses, we tested the association of *APOE*ε4 with CTE stage (0-IV; 0=no CTE) and semi-quantitative tau burden across the 11 brain regions in separate, multivariable ordinal logistic regression models. Among all brain donors and in median age-stratified analyses, we

© 2022 Atherton K et al. *JAMA Neurology.*

tested the association of *APOE*ε4 with quantitative tau burden in the dorsal lateral frontal lobe in linear regression models. To test whether the *APOE*ε4 association was independent of an AD Reagan neuropathological diagnosis (i.e. intermediate or high likelihood of dementia due to AD) and Aβ pathology, we conducted sensitivity analyses excluding donors with an AD neuropathological diagnosis and further adjusting for measures of neuritic and diffuse plaques. To compare the relative association sizes of *APOE*ε4 with age and duration of play among football players, we repeated the above regression models among all brain donors who played football, adding a duration of play term in years. To test whether there was gene-environment interaction with duration of football play, we added a duration of play by ε4 interaction term. We conducted additional sensitivity analyses excluding self-reported Black donors and using different age cut-offs (55 years and 75 years) for age-stratified analyses. Statistical significance was set at $\alpha \leq 0.05$ after false discovery rate (FDR) correction (4 tests for the primary outcomes: CTE, CTE stage, dementia, quantitative tau burden in the dorsal lateral frontal lobe; 11 tests for the regional semi-quantitative tau burden). Lastly, we conducted identical analyses for *APOE*ε2. Statistical analyses were performed using R (v.3.6.1).

© 2022 Atherton K et al. *JAMA Neurology.*

eTable 1: Missingness of semi-quantitative and quantitative tau burden data across brain regions

Region	Frequency (%) missing among brain donors before imputation (n=364)
Dorsolateral Frontal (semi-quantitative)	30 (8.2%)
Inferior Orbital Frontal (semi-quantitative)	40 (11.0%)
Superior Temporal (semi-quantitative)	40 (11.0%)
Inferior Parietal (semi-quantitative)	43 (11.8%)
CA1 hippocampal subfield (semi-quantitative)	26 (7.1%)
CA2 hippocampal subfield (semi-quantitative)	44 (12.1%)
CA4 hippocampal subfield (semi-quantitative)	33 (9.1%)
Entorhinal Cortex (semi-quantitative)	28 (7.7%)
Amygdala (semi-quantitative)	32 (8.8%)
Substantia Nigra (semi-quantitative)	28 (7.7%)
Locus Coeruleus (semi-quantitative)	39 (10.7%)
Dorsolateral Frontal Lobe (quantitative)	26 (7.1%)

© 2022 Atherton K et al. *JAMA Neurology.*

eTable 2. Demographic, clinical, head trauma-related, genetic and neuropathological characteristics stratified by CTE control status and CTE Stage among donors age>65 years.

	Total (n=181)	Controls (n=25)	CTE (n=156)	Stage I (n=7)	Stage II (n=19)	Stage III (n=49)	Stage IV (n=81)
Demographic and clinical							
**Age, median, (IQR), (range)**	77.0, (71.0-82.0), (66-98)	77.0, (71.5-81.5), (66-89)	77.0, (71.0-82.0), (66-98)	84.0, (75.0-88.0), (71-89)	73.0, (68.0-78.0), (66-89)	76.0, (70.5-80.0), (66-89)	78.0, (72.5-83.0), (66-98)
**Self-reported black race, n (%)**	19 (10.5)	1 (4.0)	18 (11.5)	0	2 (10.5)	9 (18.4)	7 (8.6)
**Dementia, n (%)**	148 (82.2)	16 (64.0)	132 (84.6)	4 (57.0)	14 (73.7)	37 (75.5)	77 (95.1)
**Cognitive symptoms present, n (%)**	172 (95.0)	20 (80.0)	152 (97.4)	5 (71.4)	19 (100.0)	47 (95.9)	81 (100.0)
**Cause of death**							
**Suicide, n (%)**	4 (2.2)	0	4 (2.6)	0	2 (10.5)	1 (2.0)	1 (1.2)
**Accidental overdose, n (%)**	0	0	0	0	0	0	0
**Cardiovascular disease, n (%)**	25 (13.8)	1 (4.0)	24 (15.4)	1 (14.3)	4 (21.1)	13 (26.5)	6 (7.4)
**Neurodegenerative disease, n (%)**	109 (60.2)	14 (56.0)	95 (60.9)	3 (42.9)	9 (47.4)	21 (42.9)	62 (76.5)
**Motor neuron disease, n (%)**	4 (2.2)	1 (4.0)	3 (1.9)	0	0	3 (6.1)	0
**Cancer, n (%)**	15 (8.3)	2 (8.0)	13 (8.3)	0	1 (5.3)	7 (14.3)	5 (6.2)
**Injury, n (%)**	2 (1.1)	0	2 (1.3)	0	0	0	2 (2.5)
**Other, n (%)**	22 (12.2)	7 (28.0)	17 (10.9)	3 (42.9)	3 (15.8)	4 (8.2)	5 (6.2)
**Unknown, n (%)**	0	0	0	0	0	0	0
Head trauma-related							
**Contact sports, n (%)**	173 (95.6)	17 (68.0)	156 (100.0)	7 (100.0)	19 (100.0)	49 (100.0)	81 (100.0)
**Age of First Exposure to Contact**	13.1 ± 2.8 (5-25)	11.6 ± 2.8 (7-16)	13.3 ± 2.7 (6-25)	14.7 ± 1.0 (13-16)	13.5 ± 2.4 (8-16)	13.3 ± 2.7 (6-20)	12.9 ± 2.5 (6-20)

© 2022 Atherton K et al. *JAMA Neurology.*

**Sports, mean ± SD (range)**							
**Football, n (%)**	163 (90.1)	16 (64.0)	147 (94.2)	6 (85.7)	17 (89.5)	48 (98.0)	76 (93.8)
**Professional highest level, n (%)**	107 (59.1)	4 (16.0)	103 (66.0)	3 (42.9)	12 (63.2)	34 (69.4)	54 (66.7)
**College/semi-professional highest level, n (%)**	44 (24.3)	4 (16.0)	40 (25.6)	1 (14.3)	5 (26.3)	13 (26.5)	21 (25.9)
**High School/youth highest level, n (%)**	12 (6.6)	8 (32.0)	4 (2.6)	2 (28.6)	0	1 (2.0)	1 (1.2)
**Years of Football, mean ± SD (range)**	13.9 ± 2.8 (1-33)	9.3 ± 6.9 (1-21)	14.5 ± 5.6 (2-33)	8.3 ± 5.4 (2-15)	12.1 ± 3.3 (6-23)	14.4 ± 5.1 (4-27)	15.1 ± 5.4 (4-33)
**Hockey, n (%)**	3 (1.7)	0	3 (1.9)	1 (14.3)	1 (5.3)	1 (2.0)	0
**Soccer, n (%)**	1 (0.6)	1 (4.0)	0	0	0	0	0
**Amateur Wrestling, n (%)**	0	0	0	0	0	0	0
**Boxing, n (%)**	4 (2.2)	0	4 (2.6)	0	0	0	4 (4.9)
**Rugby, n (%)**	2 (1.1)	0	2 (1.3)	0	1 (5.3)	0	1 (1.2)
**Other contact sports, n (%)**	49 (27.1)	6 (24.0)	43 (27.6)	4 (57.1)	6 (31.6)	14 (28.6)	19 (23.5)
**Military, n (%)**	79 (43.6)	13 (52.0)	66 (42.3)	5 (71.4)	5 (26.3)	19 (38.8)	37 (45.7)
**Combat, n (%)**	8 (4.4)	3 (12.0)	5 (3.2)	1 (14.3)	0	0	4 (4.9)
APOE							
**ε2 carriers, n (%)**	24 (13.3)	3 (12.0)	21 (13.5)	1 (14.3)	3 (15.8)	19 (38.8)	12 (14.8)
**ε4 carriers, n (%)**	68 (37.6)	8 (32.0)	60 (38.5)	0	6 (31.6)	14 (28.6)	40 (49.4)
**ε2ε2, n (%)**	2 (1.1)	0	2 (1.3)	0	1 (5.3)	0	1 (1.2)
**ε2ε3, n (%)**	15 (8.3)	2 (8.0)	13 (8.3)	1 (14.3)	2 (10.5)	5 (10.2)	5 (6.2)
**ε2ε4, n (%)**	7 (3.9)	1 (4.0)	6 (3.8)	0	0	0	6 (7.4)

© 2022 Atherton K et al. *JAMA Neurology.*

**ε3ε3, n (%)**	96 (53.0)	15 (60.0)	81 (51.9)	6 (85.7)	10 (52.6)	30 (61.2)	35 (43.2)
**ε3ε4, n (%)**	53 (29.3)	6 (24.0)	47 (30.1)	0	6 (31.6)	13 (26.5)	28 (34.6)
**ε4ε4, n (%)**	8 (4.4)	1 (4.0)	7 (4.5)	0	0	1 (2.0)	6 (7.4)
Pathology							
**Log quantitative tau burden in dorsolateral frontal lobe, mean ± SD (range), tau+ pixels/mm^2**	8.4 ± 1.9 (4.6-12.5)	7.2 ± 2.0 (4.9-10.8)	8.5 ± 1.9 (4.6-12.5)	6.2 ± 1.0 (4.8-7.6)	7.2 ± 1.7 (5.4-10.9)	7.6 ± 1.4 (4.6-10.4)	9.5 ± 1.6 (6.3-12.5)
**AD pathology, n (%)**	45 (24.9)	8 (32.0)	37 (23.7)	2 (28.6)	2 (10.5)	7 (14.3)	26 (32.1)
**CERAD neuritic plaque score, mean ± SD**	0.94 ± 0.94	1.00 ± 1.12	0.93 ± 0.91	0.57 ± 0.79	0.42 ± 0.61	0.71 ± 0.89	1.21 ± 0.91
**Braak NFT stage, mean ± SD**	3.34 ± 1.62	2.87 ± 2.05	3.41 ± 1.54	2.57 ± 1.62	2.63 ± 1.12	3.15 ± 1.44	3.85 ± 1.56
**Lewy body pathology, n (%)**	50 (27.6)	9 (36.0)	41 (26.3)	3 (42.9)	3 (15.8)	14 (28.6)	21 (25.9)
**Brainstem predominant, n (%)**	30 (16.6)	4 (16.0)	26 (16.7)	1 (14.3)	1 (5.3)	8 (16.3)	16 (19.8)
**Limbic/neocortical predominant, n (%)**	20 (11.0)	5 (20.0)	15 (9.6)	2 (28.6)	2 (10.5)	6 (12.2)	5 (6.2)
**FTLD tau, n (%)**	16 (8.8)	4 (16.0)	12 (7.7)	1 (14.3)	3 (15.8)	4 (8.2)	4 (4.9)
**FTLD tdp-43, n (%)**	15 (8.3)	1 (4.0)	14 (9.0)	1 (14.3)	2 (10.5)	1 (2.0)	10 (12.3)

Abbreviations: CERAD: Consortium to Establish a Registry for Alzheimer's Disease; FTLD: frontotemporal lobar degeneration; NFT: neurofibrillary tangle

© 2022 Atherton K et al. *JAMA Neurology.*

eTable 3. Demographic, clinical, head trauma-related, genetic and neuropathological characteristics stratified by CTE control status and CTE Stage among donors age≤65 years.

	Total (n=183)	Controls (n=45)	CTE (n=138)	Stage I (n=35)	Stage II (n=44)	Stage III (n=47)	Stage IV (n=12)
Demographic and clinical							
**Age, median, (IQR), (range)**	47.0, (31.0-59.0), (20-65)	40.0, (28.5-55.5), (20-65)	48.5, (32.0-60.0), (20-65)	29.0, (24.0-48.0), (20-65)	44.5, (30.3-55.5), (21-65)	53.0, (47.0-61.0), (25-65)	61.5, (55.5-64.0), (46-65)
**Self-reported black race, n (%)**	34 (18.6)	5 (11.1)	29 (21.0)	6 (17.1)	5 (11.4)	15 (31.9)	3 (25.0)
**Dementia, n (%)**	57 (31.1)	11 (24.4)	46 (33.3)	9 (25.7)	7 (15.9)	20 (42.6)	10 (83.3)
**Cognitive symptoms present, n (%)**	155 (84.7)	35 (77.8)	120 (87.0)	30 (85.7)	36 (81.8)	43 (91.5)	11 (91.7)
**Cause of death**							
**Suicide, n (%)**	49 (26.8)	16 (35.6)	33 (23.9)	13 (37.1)	13 (29.5)	7 (14.9)	0
**Accidental overdose, n (%)**	18 (9.8)	5 (11.1)	13 (9.4)	1 (2.9)	6 (13.6)	6 (12.8)	0
**Cardiovascular disease, n (%)**	35 (19.1)	5 (11.1)	30 (21.7)	4 (11.4)	11 (25.0)	14 (29.8)	1 (8.3)
**Neurodegenerative disease, n (%)**	20 (10.9)	6 (13.3)	14 (10.1)	2 (5.7)	2 (4.5)	3 (6.4)	7 (58.3)
**Motor neuron disease, n (%)**	13 (7.1)	1 (2.2)	12 (8.7)	1 (2.9)	5 (11.4)	4 (8.5)	2 (16.7)
**Cancer, n (%)**	8 (4.4)	1 (2.2)	7 (5.1)	2 (5.7)	2 (4.5)	2 (4.3)	1 (8.3)
**Injury, n (%)**	6 (3.3)	2 (4.4)	4 (2.9)	2 (5.7)	0	2 (4.3)	0
**Other, n (%)**	32 (17.5)	8 (17.8)	14 (10.1)	10 (28.6)	4 (9.1)	9 (19.1)	1 (8.3)
**Unknown, n (%)**	1 (0.5)	1 (2.2)	0	0	0	0	0
Head trauma-related							
**Contact sports, n (%)**	179 (97.8)	41 (91.1)	138 (100.0)	35 (100.0)	44 (100.0)	47 (100.0)	12 (100.0)
**Age of First Exposure to Contact**	10.5 ± 3.7 (3-34)	11.9 ± 4.7 (5-34)	10.2 ± 3.1 (3-16)	10.2 ± 3.3 (4-16)	10.2 ± 2.3 (6-14)	10.8 ± 2.6 (5-15)	11.2 ± 3.5 (5-16)

© 2022 Atherton K et al. *JAMA Neurology.*

**Sports, mean ± SD (range)**							
**Football, n (%)**	160 (87.4)	36 (80.0)	124 (89.9)	30 (85.7)	37 (84.1)	47 (100.0)	10 (83.3)
**Professional highest level, n (%)**	63 (34.4)	8 (17.8)	55 (39.9)	7 (20.0)	13 (29.5)	31 (66.0)	4 (33.3)
**College/semi-professional highest level, n (%)**	61 (73.5)	10 (22.2)	51 (37.0)	13 (37.1)	18 (40.9)	14 (29.8)	6 (50.0)
**High School/youth highest level, n (%)**	36 (19.7)	18 (40.0)	18 (13.0)	10 (28.6)	6 (13.6)	2 (4.3)	0
**Years of Football, mean ± SD (range)**	11.9 ± 5.6 (1-25)	8.0 ± 3.6 (1-15)	13.0 ± 5.6 (1-25)	10.0 ± 3.6 (1-20)	12.4 ± 4.9 (3-25)	15.1 ± 5.4 (1-25)	13.5 ± 7.1 (6-25)
**Hockey, n (%)**	10 (5.5)	2 (4.4)	8 (5.8)	3 (8.6)	4 (9.1)	0	1 (8.3)
**Soccer, n (%)**	5 (2.7)	1 (2.2)	4 (2.9)	2 (5.7)	2 (4.5)	0	0
**Amateur Wrestling, n (%)**	2 (1.1)	2 (4.4)	0	0	0	0	0
**Boxing, n (%)**	2 (1.1)	0	2 (1.4)	1 (2.9)	0	0	1 (8.3)
**Rugby, n (%)**	2 (1.1)	0	2 (1.4)	0	1 (2.3)	0	1 (8.3)
**Other contact sports, n (%)**	65 (35.5)	20 (44.4)	45 (32.6)	15 (42.9)	17 (38.6)	8 (17.0)	5 (41.7)
**Military, n (%)**	18 (9.8)	8 (17.8)	10 (7.2)	5 (14.3)	3 (6.8)	0	2 (16.7)
**Combat, n (%)**	12 (6.6)	5 (11.1)	7 (5.1)	3 (8.6)	3 (6.8)	0	1 (8.3)
APOE							
**ε2 carriers, n (%)**	18 (9.8)	5 (11.1)	13 (9.4)	3 (8.6)	4 (9.1)	5 (10.6)	1 (8.3)
**ε4 carriers, n (%)**	60 (32.8)	12 (26.7)	48 (34.8)	12 (34.3)	12 (27.3)	18 (38.3)	6 (50.0)
**ε2ε2, n (%)**	1 (0.5)	1 (2.2)	0	0	0	0	0
**ε2ε3, n (%)**	14 (7.7)	3 (6.7)	11 (8.0)	3 (8.6)	3 (6.8)	4 (8.5)	1 (8.3)
**ε2ε4, n (%)**	3 (1.6)	1 (2.2)	2 (1.4)	0	1 (2.3)	1 (2.1)	0

© 2022 Atherton K et al. *JAMA Neurology.*

**ε3ε3, n (%)**	108 (59.0)	29 (64.4)	79 (57.2)	20 (57.1)	29 (65.9)	25 (53.2)	5 (41.7)
**ε3ε4, n (%)**	49 (26.8)	9 (20.0)	40 (29.0)	10 (28.6)	11 (25.0)	14 (29.8)	5 (41.7)
**ε4ε4, n (%)**	8 (4.4)	2 (4.4)	6 (4.3)	2 (5.7)	0	3 (6.4)	1 (8.3)
Pathology							
**Log quantitative tau burden in dorsolateral frontal lobe, mean ± SD (range), tau+ pixels/mm^2**	6.2 ± 1.9 (3.3-13.3)	5.4 ± 1.3 (3.5-10.0)	6.5 ± 2.0 (3.3-13.3)	5.1 ± 0.8 (3.3-6.5)	5.8 ± 1.5 (3.6-12.6)	7.1 ± 1.4 (5.0-11.4)	10.6 ± 1.4 (8.9-13.3)
**AD pathology, n (%)**	10 (5.5)	4 (8.9)	6 (4.3)	0	1 (2.3)	1 (2.1)	4 (33.3)
**CERAD neuritic plaque score, mean ± SD**	0.28 ± 0.95	0.27 ± 0.81	0.28 ± 1.00	0.06 ± 0.24	0.39 ± 1.48	0.15 ± 0.52	1.08 ± 1.24
**Braak NFT stage, mean ± SD**	1.46 ± 2.01	0.96 ± 2.13	1.62 ± 1.95	0.36 ± 0.78	0.82 ± 1.26	2.69 ± 1.69	4.27 ± 2.76
**Lewy body pathology, n (%)**	13 (7.1)	2 (4.4)	11 (8.0)	2 (5.7)	2 (4.5)	3 (6.4)	4 (33.3)
**Brainstem predominant, n (%)**	7 (3.8)	0	7 (5.1)	2 (5.7)	2 (4.5)	0	3 (25.0)
**Limbic/neocortical predominant, n (%)**	6 (3.3)	2 (4.4)	4 (2.9)	0	0	3 (6.4)	1 (8.3)
**FTLD tau, n (%)**	6 (3.3)	2 (4.4)	4 (2.9)	1 (2.9)	0	1 (2.1)	2 (16.7)
**FTLD tdp-43, n (%)**	4 (2.2)	1 (2.2)	3 (2.2)	1 (2.9)	1 (2.3)	0	1 (8.3)

Abbreviations: CERAD: Consortium to Establish a Registry for Alzheimer's Disease; FTLD: frontotemporal lobar degeneration; NFT: neurofibrillary tangle

© 2022 Atherton K et al. *JAMA Neurology.*

eTable 4. Estimated associations of *APOE* ε4 status on CTE diagnosis, CTE stage, dementia diagnosis, and quantitative tau burden in the dorsolateral frontal lobe among self-reported White race, stratified by age 65 and excluding donors meeting AD Reagan criteria.

	Age ≤ 65 (n=149)			Age > 65 (n=162)			Age >65; excluding donors meeting AD Reagan criteria (n=117)		
**Outcome**	**OR**	**95% CI**	**FDR-corrected p-value**	**OR**	**95% CI**	**FDR-corrected p-value**	**OR**	**95% CI**	**FDR-corrected p-value**
**CTE Diagnosis**	1.21	0.54-2.72	0.75	1.19	0.47-2.98	0.75	1.44	0.43-4.84	0.75
**CTE Stage**[a]	1.18	0.63-2.21	0.75	2.20	1.18-4.09	0.04	3.00	1.37-6.57	0.03
**dementia**	1.16	0.46-2.93	0.75	3.50	1.24-9.86	0.05	3.32	1.02-10.74	0.10
	**beta**	**95% CI**	**FDR-corrected p-value**	**beta**	**95% CI**	**FDR-corrected p-value**	**beta**	**95% CI**	**FDR-corrected p-value**
**Quantitative tau burden in dorsolateral frontal lobe (log tau+ pixels/mm²)**[b]	0.54	-0.11-1.18	0.18	1.48	0.88-2.08	3.40×10^{-5}	1.12	0.50-1.74	3.34×10^{-3}

All analyses are adjusted for age.

[a]Odds ratio is the odds of increasing 1 stage (0-4 scale) for ε4 carriers compared with non-carriers

[c]Beta value is the increase in log tau+ pixels/mm² in the dorsolateral frontal lobe for ε4 carriers compared with non-carriers

© 2022 Atherton K et al. *JAMA Neurology.*

eTable 5. Estimated associations of *APOE* ε4 status on CTE diagnosis, CTE stage, dementia diagnosis, and quantitative tau burden in the dorsolateral frontal lobe, among the older age group, using different age-cut points

	Age > 55 (n=241)			Age > 65 (n=181)			Age >75 (n=104)		
**Outcome**	**OR**	**95% CI**	**FDR-corrected p-value**	**OR**	**95% CI**	**FDR-corrected p-value**	**OR**	**95% CI**	**FDR-corrected p-value**
**CTE Diagnosis**	1.15	0.55-2.44	0.77	1.30	0.53-3.22	0.57	1.07	0.32-3.58	0.92
**CTE Stage**[a]	1.88	1.16-3.06	0.02	2.34	1.30-4.20	0.01	3.08	1.33-7.12	0.02
**dementia**	2.14	1.08-4.26	0.05	2.64	1.06-6.61	0.08	4.99	1.02-24.4	0.07
	**beta**	**95% CI**	**FDR-corrected p-value**	**beta**	**95% CI**	**FDR-corrected p-value**	**beta**	**95% CI**	**FDR-corrected p-value**
**Quantitative tau burden in dorsolateral frontal lobe (log tau+ pixels/mm²)**[b]	1.23	0.73-1.74	$1.59x10^{-5}$	1.39	0.83-1.94	$2.37x10^{-5}$	1.74	1.04-2.43	$1.59x10^{-5}$

All analyses are adjusted for age.

[a]Odds ratio is the odds of increasing 1 stage (0-4 scale) for ε4 carriers compared with non-carriers

[c]Beta value is the increase in log tau+ pixels/mm² in the dorsolateral frontal lobe for ε4 carriers compared with non-carriers

© 2022 Atherton K et al. *JAMA Neurology.*

eTable 6: Estimated associations of *APOE* ε4 status on semi-quantitative tau burden in brain regions commonly affected in CTE, stratified by age 65 and excluding donors meeting AD Reagan criteria.

	Age ≤ 65 (n=183)			Age > 65 (n=181)			Age >65; Excluding donors meeting AD Reagan criteria (n=136)		
**Region**	**OR**	**95% CI**	**FDR-corrected p-value**	**OR**	**95% CI**	**FDR-corrected p-value**	**OR**	**95% CI**	**FDR-corrected p-value**
**Inferior Parietal**	1.36	0.75-2.47	0.41	3.26	1.82-5.84	$3.48x10^{-3}$	2.69	1.39-5.26	0.03
**Inferior Orbital Frontal**	1.76	0.96-3.25	0.17	2.93	1.62-5.31	0.01	2.47	1.27-4.94	0.06
**Dorsolateral Frontal**	1.55	0.86-2.79	0.25	2.93	1.59-5.38	0.01	2.17	1.14-4.28	0.10
**Amygdala**	1.67	0.89-3.14	0.23	3.08	1.63-5.80	0.01	2.06	1.04-4.17	0.14
**Entorhinal Cortex**	1.70	0.91-3.19	0.22	2.45	1.27-4.75	0.05	2.01	1.00-4.21	0.17
**CA4 hippocampal subfield**	1.87	0.98-3.57	0.17	1.53	0.87-2.67	0.25	2.01	1.00-3.97	0.14
**Superior Temporal**	1.09	0.59-2.01	0.80	2.12	1.15-3.91	0.07	1.70	0.87-3.46	0.25
**CA1 hippocampal subfield**	1.63	0.86-3.11	0.25	1.44	0.82-2.53	0.30	1.15	0.57-2.23	0.75
**CA2 hippocampal subfield**	1.57	0.80-3.08	0.29	1.17	0.67-2.04	0.68	1.14	0.55-2.21	0.76
**Substantia Nigra**	0.98	0.52-1.86	0.96	1.46	0.84-2.53	0.28	1.41	0.71-2.72	0.41
**Locus Coeruleus**	1.29	0.71-2.34	0.50	1.30	0.72-2.32	0.49	1.17	0.60-2.31	0.74

All analyses were adjusted for age and race. Odds ratio is the odds of increasing 1 level (0-3 scale) for ε4 carriers compared with non-carriers.

© 2022 Atherton K et al. *JAMA Neurology.*

eTable 7: Estimated associations of *APOE* ε4 status when adjusting for Aβ pathology among donors older than 65 years, with and without exclusion of donors meeting AD Reagan criteria

	**beta**	**95% CI**	**AIC**	**r^2**
Original Model	1.39	0.83-1.94	731	0.15
Adjusting for Neuritic Plaques	0.85	0.35-1.35	676	0.36
Adjusting for Diffuse Plaques	0.58	0.04-1.11	683	0.34

All analyses were adjusted for age and self-reported race.
Beta value is the increase in log tau+ pixels/mm^2 in the dorsolateral frontal lobe for ε4 carriers compared with non-carriers

© 2022 Atherton K et al. *JAMA Neurology.*

eTable 8: Estimated associations of *APOE* ε4 status, years of football played and their interaction; Age>65

	Years played Marginal Term			*APOE* ε4 Marginal Term			Interaction Term		
**Outcome**	**OR**	**95% CI**	**FDR corrected p-value**	**OR**	**95% CI**	**FDR corrected p-value**	**OR**	**95% CI**	**FDR corrected p-value**
**CTE Diagnosis**	1.39	1.16-1.66	$9.6x10^{-4}$	7.76	0.54-112	0.13	0.83	0.65-1.06	0.17
**CTE Stage**	1.20	1.12-1.29	$3.4x10^{-6}$	10.5	1.78-62	0.02	0.92	0.81-1.04	0.17
**Dementia**	1.11	1.01-1.21	0.85	30.4	0.96-959	0.07	0.85	0.70-1.04	0.17
	**Beta**	**95% CI**	**FDR corrected p-value**	**Beta**	**95% CI**	**FDR corrected p-value**	**Beta**	**95% CI**	**FDR corrected p-value**
**Quantitative tau burden in dorsolateral frontal lobe (log tau+ pixels/mm^2)**	0.09	0.03-0.15	0.003	2.84	1.36-4.32	$9.6x10^{-4}$	-0.10	-0.20-0.01	0.16

Analyses limited to football players, Age>65 (n=163)
All analyses were adjusted for age and self-reported race.

© 2022 Atherton K et al. *JAMA Neurology.*

eTable 9: Estimated associations of *APOE* ε4 status, years of football played and their interaction; Age≤65

	Years played Marginal Term			*APOE* ε4 Marginal Term			Interaction Term		
**Outcome**	**OR**	**95% CI**	**FDR corrected p-value**	**OR**	**95% CI**	**FDR corrected p-value**	**OR**	**95% CI**	**FDR corrected p-value**
**CTE Diagnosis**	1.23	1.09-1.38	0.002	0.42	0.04-4.40	0.69	1.07	0.86-1.33	0.99
**CTE Stage**	1.18	1.10-1.27	$6.5x10^{-5}$	1.04	0.21-5.11	0.96	0.99	0.88-1.10	0.99
**Dementia**	1.12	1.02-1.24	0.03	19.2	2.19-169.3	0.03	0.78	0.66-0.91	0.01
	**Beta**	**95% CI**	**FDR corrected p-value**	**Beta**	**95% CI**	**FDR corrected p-value**	**Beta**	**95% CI**	**FDR corrected p-value**
**Quantitative tau burden in dorsolateral frontal lobe (log tau+ pixels/mm^2)**	0.06	0.00-0.13	0.06	0.47	-0.95-1.89	0.69	0.00	-0.10-0.10	0.99

Analyses limited to football players, Age≤65 (n=160)
All analyses were adjusted for age and self-reported race.

© 2022 Atherton K et al. *JAMA Neurology.*

eTable 10. Estimated associations of *APOE* ε2 status on CTE diagnosis, CTE stage, dementia diagnosis, and quantitative tau burden in the dorsolateral frontal lobe stratified by median age 65 and overall.

	Age ≤ 65 (n=183)			Age > 65 (n=181)			All (n=364)		
**Outcome**	**OR**	**95% CI**	**unadjusted p-value**	**OR**	**95% CI**	**unadjusted p-value**	**OR**	**95% CI**	**unadjusted p-value**
CTE Diagnosis	0.74	0.24-2.25	0.60	1.17	0.32-4.26	0.82	0.90	0.39-2.08	0.81
CTE Stage[a]	0.79	0.32-1.94	0.60	1.17	0.52-2.63	0.70	1.01	0.56-1.83	0.97
dementia	0.91	0.25-3.35	0.89	1.68	0.46-6.13	0.43	1.26	0.53-3.02	0.60
	**beta**	**95% CI**	**unadjusted p-value**	**beta**	**95% CI**	**unadjusted p-value**	**beta**	**95% CI**	**unadjusted p-value**
Quantitative tau burden in dorsolateral frontal lobe (log tau+ pixels/mm^2)[b]	0.10	-0.75-0.96	0.82	0.46	-0.37-1.29	0.28	0.33	-0.26-0.93	0.28

All analyses are adjusted for age and self-reported race.
[a]Odds ratio is the odds of increasing 1 stage (0-4 scale) for ε2 carriers compared with non-carriers
[c]Beta value is the increase in log tau+ pixels/mm^2 in the dorsolateral frontal lobe for ε2 carriers compared with non-carriers

© 2022 Atherton K et al. *JAMA Neurology.*

eTable 11: Estimated associations of *APOE* ε2 status on semi-quantitative tau burden in brain regions commonly affected in CTE, stratified by median age 65 and overall

	Age ≤ 65 (n=183)			Age > 65 (n=181)			All (n=364)		
**Region**	**OR**	**95% CI**	**unadjusted p-value**	**OR**	**95% CI**	**unadjusted p-value**	**OR**	**95% CI**	**unadjusted p-value**
Inferior Parietal	0.78	0.30-2.03	0.61	1.28	0.58-2.84	0.54	1.06	0.58-1.94	0.86
Inferior Orbital Frontal	0.95	0.37-2.47	0.92	0.82	0.37-1.82	0.62	0.89	0.48-1.63	0.70
Dorsolateral Frontal	0.75	0.30-1.92	0.55	1.00	0.44-2.27	1.00	0.88	0.48-1.62	0.67
Amygdala	1.54	0.60-3.93	0.37	0.68	0.32-1.46	0.33	0.98	0.53-1.79	0.94
Entorhinal Cortex	1.55	0.59-4.05	0.38	0.47	0.21-1.06	0.07	0.81	0.42-1.53	0.51
CA4 hippocampal subfield	1.37	0.51-3.71	0.53	0.61	0.27-1.35	0.22	0.90	0.48-1.68	0.74
Superior Temporal	0.75	0.30-1.89	0.55	0.79	0.36-1.73	0.56	0.80	0.45-1.45	0.47
CA1 hippocampal subfield	0.97	0.37-2.57	0.96	1.04	0.45-2.41	0.92	1.05	0.57-1.96	0.87
CA2 hippocampal subfield	2.33	0.82-6.62	0.11	1.20	0.54-2.67	0.66	1.64	0.88-3.05	0.12
Substantia Nigra	1.72	0.64-4.63	0.29	1.04	0.47-2.28	0.93	1.28	0.69-2.38	0.44
Locus Coeruleus	0.98	0.36-2.65	0.97	0.95	0.42-2.15	0.91	0.97	0.51-1.81	0.92

All analyses were adjusted for age and race.
Odds ratio is the odds of increasing 1 level (0-3 scale) for each additional ε2 allele.

© 2022 Atherton K et al. *JAMA Neurology.*

eFigure 1: Flow chart of included and excluded brain donors

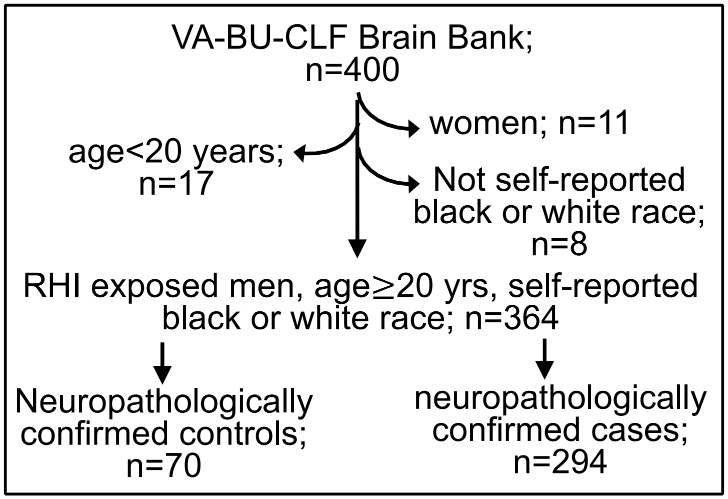

© 2022 Atherton K et al. *JAMA Neurology.*

eFigure 2. Actual and predicted values from multiple imputation for each neuropathological region

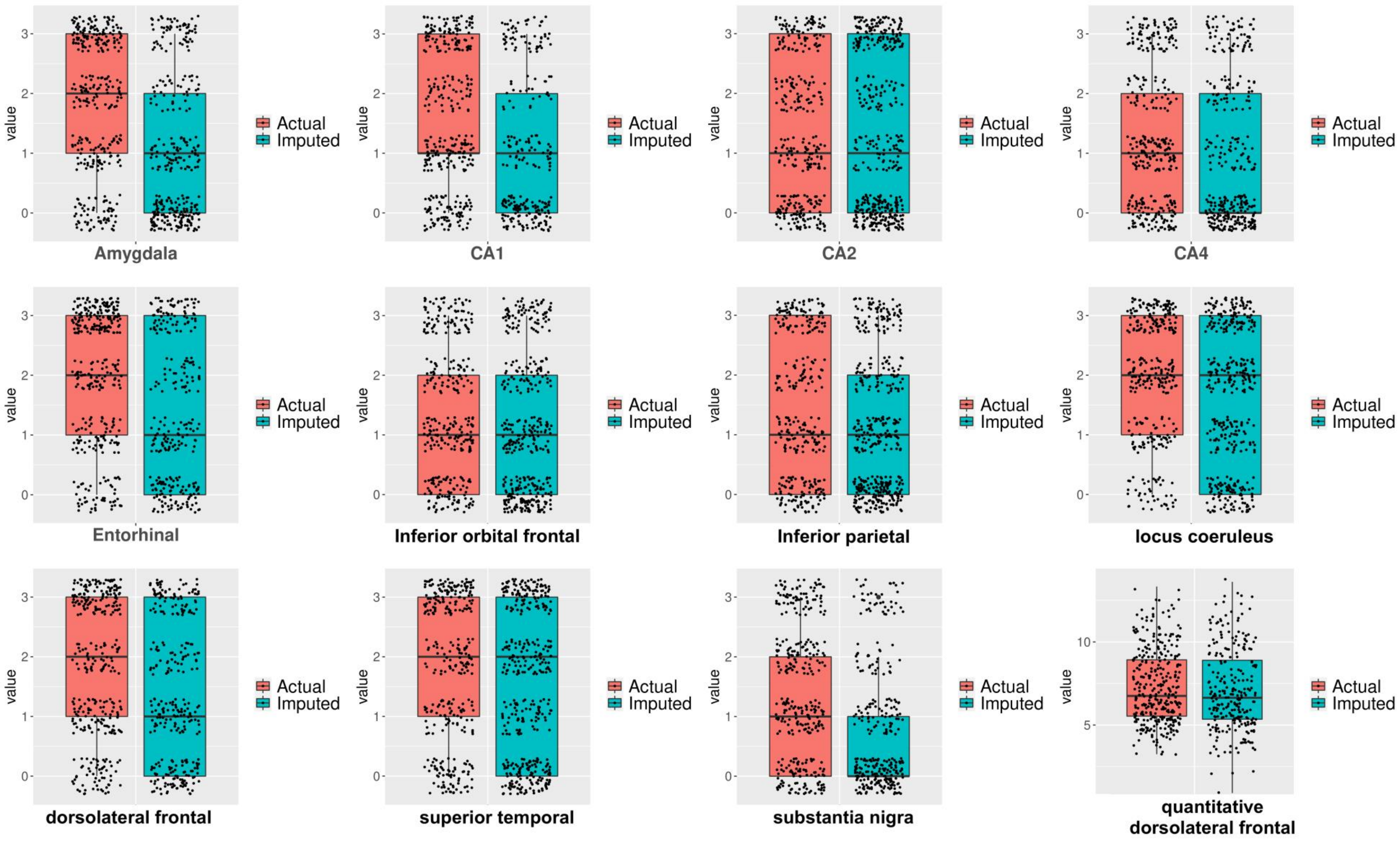

© 2022 Atherton K et al. *JAMA Neurology.*

eReferences

1. Little J, Higgins JPT, Ioannidis JPA, et al. STrengthening the REporting of Genetic Association Studies (STREGA)—an extension of the STROBE statement. *Genet Epidemiol*. 2009;33(7):581-598. doi:10.1002/gepi.20410

2. *Diagnostic and Statistical Manual of Mental Disorders DSM-IV-TR Fourth Edition*. Diagnostic and statistical manual of mental disorders (4th ed., Text Revision); 2000.

3. Vonsattel JPG, Amaya M del P, Cortes EP, Mancevska K, Keller CE. 21st Century Brain Banking Practical prerequisites and lessons from the past: The experience of New York Brain Bank – Taub Institute - Columbia University. *Cell Tissue Bank*. 2008;9(3):247-258. doi:10.1007/s10561-008-9079-y

4. Vonsattel JPG, del Amaya MP, Keller CE. Twenty-first century brain banking. Processing brains for research: the Columbia University methods. *Acta Neuropathol (Berl)*. 2008;115(5):509-532. doi:10.1007/s00401-007-0311-9

5. McKee AC, Cantu RC, Nowinski CJ, et al. Chronic Traumatic Encephalopathy in Athletes: Progressive Tauopathy following Repetitive Head Injury. *J Neuropathol Exp Neurol*. 2009;68(7):709-735. doi:10.1097/NEN.0b013e3181a9d503

6. Vonsattel JP, Aizawa H, Ge P, et al. An improved approach to prepare human brains for research. *J Neuropathol Exp Neurol*. 1995;54(1):42-56.

7. McKee AC, Cairns NJ, Dickson DW, et al. The first NINDS/NIBIB consensus meeting to define neuropathological criteria for the diagnosis of chronic traumatic encephalopathy. *Acta Neuropathol (Berl)*. 2016;131(1):75-86. doi:10.1007/s00401-015-1515-z

8. McKee AC, Stern RA, Nowinski CJ, et al. The spectrum of disease in chronic traumatic encephalopathy. *Brain J Neurol*. 2013;136(Pt 1):43-64. doi:10.1093/brain/aws307

9. Newell KL, Hyman BT, Growdon JH, Hedley-Whyte ET. Application of the National Institute on Aging (NIA)-Reagan Institute criteria for the neuropathological diagnosis of Alzheimer disease. *J Neuropathol Exp Neurol*. 1999;58(11):1147-1155.

10. McKeith IG. Consensus guidelines for the clinical and pathologic diagnosis of dementia with Lewy bodies (DLB): report of the Consortium on DLB International Workshop. *J Alzheimers Dis JAD*. 2006;9(3 Suppl):417-423.

11. Scolding null. Greenfield’s neuropathology. Sixth edition. *J Neurol Neurosurg Psychiatry*. 1999;66(5):696.

12. Braak H, Del Tredici K. Invited Article: Nervous system pathology in sporadic Parkinson disease. *Neurology*. 2008;70(20):1916-1925. doi:10.1212/01.wnl.0000312279.49272.9f

13. Hyman BT, Phelps CH, Beach TG, et al. National Institute on Aging-Alzheimer’s Association guidelines for the neuropathologic assessment of Alzheimer’s disease. *Alzheimers Dement J Alzheimers Assoc*. 2012;8(1):1-13. doi:10.1016/j.jalz.2011.10.007

14. Montine TJ, Phelps CH, Beach TG, et al. National Institute on Aging-Alzheimer’s Association guidelines for the neuropathologic assessment of Alzheimer’s disease: a

© 2022 Atherton K et al. *JAMA Neurology.*

practical approach. *Acta Neuropathol (Berl)*. 2012;123(1):1-11. doi:10.1007/s00401-011-0910-3

15. Braak H, Braak E. Neuropathological stageing of Alzheimer-related changes. *Acta Neuropathol (Berl)*. 1991;82(4):239-259.

16. Dickson DW. Neuropathology of non-Alzheimer degenerative disorders. *Int J Clin Exp Pathol*. 2009;3(1):1-23.

17. Mackenzie IRA, Neumann M, Bigio EH, et al. Nomenclature and nosology for neuropathologic subtypes of frontotemporal lobar degeneration: an update. *Acta Neuropathol (Berl)*. 2010;119(1):1-4. doi:10.1007/s00401-009-0612-2

18. Braak H, Braak E, Bohl J. Staging of Alzheimer-related cortical destruction. *Eur Neurol*. 1993;33(6):403-408.

19. Cairns NJ, Neumann M, Bigio EH, et al. TDP-43 in familial and sporadic frontotemporal lobar degeneration with ubiquitin inclusions. *Am J Pathol*. 2007;171(1):227-240. doi:10.2353/ajpath.2007.070182

20. Brownell B, Oppenheimer DR, Hughes JT. The central nervous system in motor neurone disease. *J Neurol Neurosurg Psychiatry*. 1970;33(3):338-357.

21. Mirra SS, Heyman A, McKeel D, et al. The Consortium to Establish a Registry for Alzheimer's Disease (CERAD). Part II. Standardization of the neuropathologic assessment of Alzheimer's disease. *Neurology*. 1991;41(4):479-486.

22. Bigio EH. Update on recent molecular and genetic advances in frontotemporal lobar degeneration. *J Neuropathol Exp Neurol*. 2008;67(7):635-648. doi:10.1097/NEN.0b013e31817d751c

23. Litvan I, Hauw JJ, Bartko JJ, et al. Validity and reliability of the preliminary NINDS neuropathologic criteria for progressive supranuclear palsy and related disorders. *J Neuropathol Exp Neurol*. 1996;55(1):97-105.

24. Del Tredici K, Rüb U, De Vos RAI, Bohl JRE, Braak H. Where does parkinson disease pathology begin in the brain? *J Neuropathol Exp Neurol*. 2002;61(5):413-426.

25. Cherry JD, Tripodis Y, Alvarez VE, et al. Microglial neuroinflammation contributes to tau accumulation in chronic traumatic encephalopathy. *Acta Neuropathol Commun*. 2016;4(1):112. doi:10.1186/s40478-016-0382-8

26. Alosco ML, Cherry JD, Huber BR, et al. Characterizing tau deposition in chronic traumatic encephalopathy (CTE): utility of the McKee CTE staging scheme. *Acta Neuropathol (Berl)*. 2020;140(4):495-512. doi:10.1007/s00401-020-02197-9

27. Mez J, Daneshvar DH, Abdolmohammadi B, et al. Duration of American Football Play and Chronic Traumatic Encephalopathy. *Ann Neurol*. 2020;87(1):116-131. doi:10.1002/ana.25611

28. Masellis M, Sherborn K, Neto PR, et al. Early-onset dementias: diagnostic and etiological considerations. *Alzheimers Res Ther*. 2013;5(1):S7. doi:10.1186/alzrt197

© 2022 Atherton K et al. *JAMA Neurology.*
